# Relationship among Sugars, Organic Acids, Mineral Composition, and Chilling Injury Sensitivity on Six Pomegranate Cultivars Stored at 2 °C

**DOI:** 10.3390/foods12071364

**Published:** 2023-03-23

**Authors:** José Manuel Lorente-Mento, Alberto Carrión-Antolí, Fabián Guillén, María Serrano, Daniel Valero, Domingo Martínez-Romero

**Affiliations:** 1 Department of Applied Biology, EPSO, CIAGRO, University Miguel Hernández, Ctra. Beniel km. 3.2, 03312 Orihuela, Alicante, Spain; 2 Department of Food Technology, EPSO, CIAGRO, University Miguel Hernández, Ctra. Beniel km. 3.2, 03312 Orihuela, Alicante, Spain

**Keywords:** postharvest, *Punica granatum*, chilling injury, cold storage, sensitive varieties, anthocyanins, physiological disorder

## Abstract

Pomegranate is a sensitive fruit to chilling injury (CI) during storage at temperatures below 7 °C. However, sensitivity of pomegranate to CI is dependent on cultivar and exposure times to low temperatures. In this work, the sensitivity to CI of six pomegranate cultivars (*Punica granatum* L.) ‘Wonderful’, ‘Kingdom’, ‘Bigful’, ‘Acco’, ‘Purple Queen’, and ‘Mollar de Elche’, was evaluated after 30 d at 2 °C plus 2 d at 20 °C. Among cultivars, there was a great variability in the sensitivity to the appearance of CI symptoms. ‘Kingdom’ cultivar was the most CI sensitive and ‘Mollar de Elche’ cultivar was the least sensitive cultivar. CI symptoms were greater in the internal part of the skin than in the external part, although no correlation was found between ion leakage (IL) and CI severity after cold storage. However, both, external and internal CI index were correlated with the IL at harvest, with Pearson correlation of 0.63 and 0.80, respectively. In addition, this variability to CI among cultivars could also be due to composition and tissue structures in arils and peel. The solute content of the arils (anthocyanins, sugars, and organic acids, in particular citric acid), showed high correlations with CI sensitivity, with Pearson correlations (r) of 0.56 for total soluble solids, 0.87 for total acidity, 0.94 for anthocyanins, −0.94 for oxalic acid, 0.87 for citric acid, 0.62 for tartaric acid, −0.91 for malic acid, 0.8 for sucrose, and 0.71 for glucose, which can leak to the inner surface of the peel causing browning reactions. In addition, the high peel Ca/K ratio could play an important role on increasing fruit tolerance to CI, since it was negatively correlated with the internal and external CI indexes.

## 1. Introduction

Pomegranate (*Punica granatum* L.) is cultivated in different countries with warm and temperate climates. India, China, Iran, Turkey, Afghanistan, USA, Iraq, Pakistan, Syria, and Spain are the main pomegranate producers [1]. In the last years, the area of pomegranate cultivation has continuously increased, mainly due to increased interest of consumers for the beneficial health properties that pomegranate provides due to its bioactive compounds, with antioxidant properties (anthocyanins, fiber, etc.), as well as nutrients and minerals [2]. The Mediterranean weather is the most suitable for obtaining pomegranate fruits of adequate quality [3]. Mollar de Elche’ and ‘Wonderful’ are two of the most widely cultivated pomegranate cultivars around the world. They have significantly different agronomic requirements and physico-chemical characteristics. ‘Wonderful’, for instance, is known for its intense red color in peel and arils and it is particularly popular among pomegranate consumers. On the other hand, ‘Mollar de Elche’ has high quality desirable traits, including soft seeds and sweet aril taste, although the skin and aril colors are pale red [4].

However, pomegranate fruit is very sensitive to chilling injury (CI) during postharvest storage, and thus CI limits cold storage and commercialization. Usually, these damages appear after storage at temperatures below 5–7 °C [5]. There is a great varietal difference to CI, the threshold storage temperature for the appearance of CI symptoms being 5 °C for less sensitive cultivars and 7 °C for the most sensitive ones. For example, the ‘Ganesh’ cultivar is more sensitive to CI than ‘Wonderful’ and the threshold storage temperature is lower in the latter [6]. Accordingly, it is known that the appearance of CI in ‘Wonderful’ occurred at higher temperature (<7 °C) than in ‘Mollar de Elche’ cultivar (<5 °C), the latter being more resistant to suffer from CI [2]. This difference in sensitivity limits the storage temperature of some cultivars, reduces their post-harvest shelf life, and prevents quarantine treatments at low temperatures (~1 °C) to avoid the appearance of the fruit fly (*Ceratitis capitata*) and thus to be able to market them to countries free of this insect [7]. The occurrence of CI in the pomegranate fruit can differ from the internal to the external surface of fruit peel, always being greater on the internal surface [8]. Therefore, the consumer can accept fruit with a good external visual appearance, but with a large amount of internal damage.

Some factors linked to the sensitivity to CI are the harvest date, with the earliest cultivars being more sensitive than the late ones, as well as the maturity stage of the fruit at harvest [7]. Different postharvest strategies have been developed to reduce CI, such as the application of shock treatments with high temperatures [9] and adaptation to cold storage through treatments with low-temperature conditioning [7], as well as the use of edible coatings with carboxymethyl cellulose or carnauba wax [10], modified atmosphere packaging [11], or treatments using elicitors such as salicylic, oxalic, jasmonic acid and its derivatives [2,12], as well as methyl jasmonate [13], among others.

Endogenous aspects are linked to chilling injury, such as transcriptomic changes [6] and different gene expressions. Nian et al. [14] found that cold shocks to papayas before storage reduced the occurrence of CI due to an increase in antioxidant activity and related gene expression. CI tolerance is favored by metabolic pathways related to carbohydrates, phenols, and phenylpropanoids and there is a genotype-dependent adaptation to CI [15]. However, these works did not provide information about the metabolites involved in the CI damage symptoms. In this sense, in other plant species, the relationship has been reported between the content in particular metabolites and the sensitivity to CI, as in the case of carotenoids in mandarins [16], fatty acid composition in pineapple [17], or sugars and organic acids in cherry tomato [18]. The fatty acid fraction of the pomegranate peel stored at chilling temperatures has been the most analyzed factor [8,9]. These authors indicated that in fruit suffering from CI the ratio of polyunsaturated/saturated fatty acids (PUFA/SFA) decreased during storage. Furthermore, the low CI susceptibility of pomegranates was determined by the relationship between phenylalanine ammonia lyase (PAL) enzyme activity and (polyphenol oxidase) PPO activity [19]. However, there is no work evaluating the sensitivity to chilling injury of different pomegranate varieties in terms of their metabolites after storage at low temperatures.

The objective of this work was to assess the relationship between the content and the profile of sugars, organic acids, total anthocyanins, and minerals on the appearance of CI in six pomegranate cultivars after being stored at 2 °C for 30 d plus 2 d at 20 °C. This storage temperature was chosen to ensure that all pomegranate cultivars were under chilling conditions and could show different CI damage degree after one month of storage depending on their sensitivity. In addition, an in vitro test of skin sensitivity to internal CI was performed by using citric acid solution and pomegranate juice.

## 2. Materials and Methods

### 2.1. Experimental Design

To carry out this work, fruit from six cultivars of pomegranate (*Punica granatum* L.) were used, which were grown in the same commercial farm located at Albatera (Alicante, Spain). Fruits without visual defects were harvested in 2019 (‘Purple Queen’—4th September; ‘Acco’ and ‘Bigful’—September 10th; ‘Wonderful’, ‘Kingdom’ and ‘Mollar de Elche’—11th October) at the proper ripening stage for each cultivar, according to the following commercial criteria: sizes from 60 to 130 mm in diameter, fully cream-colored husk for ‘Mollar de Elche’ cultivar, and fully red coloured husk for the remaining cultivars (Table 1). Pomegranate trees were grown under similar standard agronomic practices and weather conditions. Pomegranate fruit were transported to the laboratory where fruits free of damage and physiological disorders were selected. For each cultivar, two batches of 3 replicates of 10 fruit were made. The first batch was used to measure fruit properties at harvest and the second one was stored for 30 d at 2 °C and 90% RH plus 2 d at 20 °C. In each fruit, the following parameters were measured: weight loss, external peel color, firmness of the whole fruit, CI on the external and internal peel, ion leakage (IL) and minerals in the skin at the end of the experiment. In addition, the total soluble solids (TSS), titratable acidity (TA), total anthocyanins, and individual organic acids and sugars were measured in pomegranate arils.

A second experiment was carried out to determine if there was a direct effect of the juice of the pomegranate arils and the appearance of internal browning of the husk. According to results of the 2019 year, in 2020, a CI sensitivity test on the inner surface of pomegranate peel was performed by using 20 fruit of ‘Wonderful’ and ‘Mollar de Elche’ (the most and lest CI sensitive cultivars). Each pomegranate fruit was cut into two parts through its equatorial plane and one of the halves had its arils removed. Subsequently and every 3 days, treatments were applied to the skins of the pomegranates by immersion for 25 min in the following solutions: distilled water, 1.5% citric acid, and fresh juices obtained from ‘Bigful’ and ‘Mollar de Elche’ (cultivars with the highest–lowest anthocyanins and high–low TA content in their juice respectively). After these treatments, pomegranate peels were placed in a dark cold room at 2 °C and 95% RH for 15 d, and then, the internal damage of the peels of the two pomegranate cultivars was evaluated.

### 2.2. CI Index

The CI index was evaluated individually for each pomegranate. This index was based on the percentage of peel surface affected by CI symptoms (dehydration, browning and pitting). A 5-point hedonic reference scale was used to quantify the external and internal CI index of pomegranate peel: 1 (no damage), 2 (0–10%), 3 (11–30%), 4 (31–50%), and 5 (>50%).

### 2.3. Fruit Quality Parameters

Weight loss was calculated as a ratio of the difference of weight of each fruit at harvest and at the end of the experiment and the weight of each fruit at harvest. Results were expressed as percentage of weight loss (%).

Fruit firmness was determined according to Lorente-Mento et al. [5] by using a TX-XT2i Texture Analyzer (Stable Microsystems, Godalming, UK). The force to deform the equatorial fruit diameter by 5% was calculated. Firmness results were expressed as the force-deformation ratio (N m^−1^).

CIELAB color space parameters (L*, a*, b*) were measured on external peel using a colorimeter CR200 (Konica Minolta, Japan) [12].

TSS, TA, and the determination of organic acids and sugars were measured according to Sayyari et al. [12]. Arils from 10 fruits were mixed to obtain a homogenous sample of each replicate, in which the following parameters were measured. About 25 g of arils from each sample was squeezed and filtered through a cotton cloth and the juice used to quantify TSS and TA in duplicate. TSS was determined by using a digital refractometer, Atago PR-101 (Atago Co., Ltd., Tokyo, Japan), at 20 °C, and expressed as %. One mL of the juice was diluted in 25 mL of distilled H_2_O and used to measure titratable acidity (TA) by automatic titration (785 DMP Titrino, Metrohm, Herisau, Switzerland) with 0.1 N NaOH up to pH 8.1, and results were expressed as g citric acid equivalent L^−1^ of juice.

Organic acids and sugars were quantified in duplicate in each sample on a Hewlett-Packard 1100 series High Performance Liquid Chromatography (HPLC) according to Garcia-Pastor et al. [20]. The elution system consisted of 0.1% phosphoric acid running isocratically at a flow rate of 0.5 mL min^−1^. Organic acids and sugars were eluted through a Supelco column (Supelcogel C-610H, 30 cm × 7.8 mm, Supelco Park, Bellefonte, PA, USA. Organic acids were detected by absorbance at 210 nm, and sugars by a refractive index detector. For the quantification, standard curves of organic acids and pure sugars were used. The results were expressed as g L^−1^ of juice.

Total anthocyanins were extracted and measured according to Lorente-Mento et al. [5]. For each sample, 20 g of arils was homogenized with methanol/formic acid/water (80:1:19, *v*/*v*/*v*) and centrifuged at 10,000× *g* for 10 min. Total anthocyanin concentration was determined, by reading the absorbance at 520 nm. The results were expressed as 10–3 g L^−1^ of cyanidin 3-glucoside equivalent (cyn 3-glu, molar absorption coefficient of 26,900 L cm^−1^ mol^−1^ and molecular weight of 449.2 g mol^−1^).

### 2.4. Peel Mineral Composition

Mineral composition was measured in duplicate in 0.25 g of dehydrated peel obtained from a mixture of 10 fruits of each replicate. Each sample was digested using a microwave digester (CEM Mars One) using 1% HNO_3_. After digestion of the samples, they were made up to 50 mL with distilled water and aliquots were taken to determine the macro- and microelements using an Inductively Coupled Plasma Mass Spectrometry (ICP-MS) (Shimadzu icpms-2030). Mineral quantification was carried out using standard curves for Ca, Cu, Fe, K, Mn, Na, and Zn. The results were expressed as g kg^−1^ on dry weight basis.

### 2.5. Statistical Analysis

The dependent variables quantified were analyzed by Analysis of Variance (ANOVA) (SPSS software version 11.0 for Windows, IBM, Chicago, IL, USA). When the differences were significant, the means were separated by Tukey test, with differences being considered significant at *p* < 0.05. In addition, Pearson correlation between external and internal peel CI and dependent variables at harvest were performed. Pearson correlations among dependent variables of fruit stored 30 d at 2 °C plus 2 d at 20 °C were also calculated. A bilateral significance test of the correlation coefficient was performed. In addition, non-linear regression was performed between CI and organic acids. All analyses were achieved with SPSS software version 11.0 for Windows.

## 3. Results and Discussion

**Figure 1:**
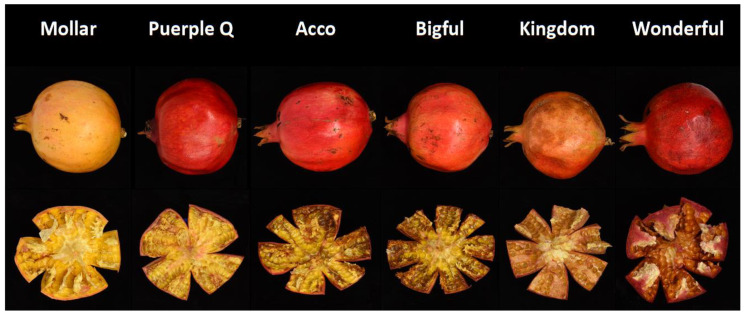
Symptoms of chilling injury on external and internal surface of skin in pomegranate cultivar after storage for 30 d at 2 °C plus 2 d at 20 °C.

All pomegranate cultivars showed CI symptoms after 30 d at 2 °C plus 2 d at 20 °C, although significant differences were observed among cultivars (Figure 1). ‘Kingdom’ and ‘Wonderful’ cultivars showed the highest internal and external CI index, followed by ‘Acco’ and ‘Bigful’ and finally, the least sensitive cultivars were ‘Purple Queen’ and ‘Mollar de Elche’.

**Figure 2:**
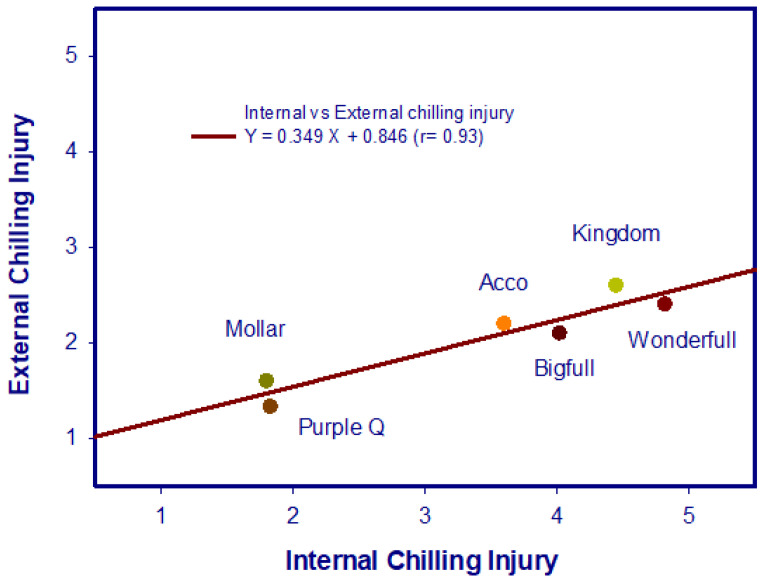
Correlation between internal and external chilling injury of six pomegranate cultivars after storage for 30 d at 2 °C plus 2 d at 20 °C.

In addition, CI on the inner peel surface was higher than on the outer one for all cultivars (Figure 1)—always keeping the proportionality of the internal and external damage in each cultivar—and they were highly correlated (r = 0.93) (Figure 2 and Table 2). Kashash et al. [15] identified several metabolic pathways that were up-regulated in pomegranate fruits exhibiting tolerance to cold temperatures. These pathways included activation of transcripts involved in jasmonic acid and ethylene hormone biosynthesis and signaling, stress-related transcription factors, calcium and mitogen-activated protein kinase signaling, starch degradation, galactinol and raffinose biosynthesis, lipid metabolism, phenol biosynthesis, and heat-shock proteins.

**Figure 3:**
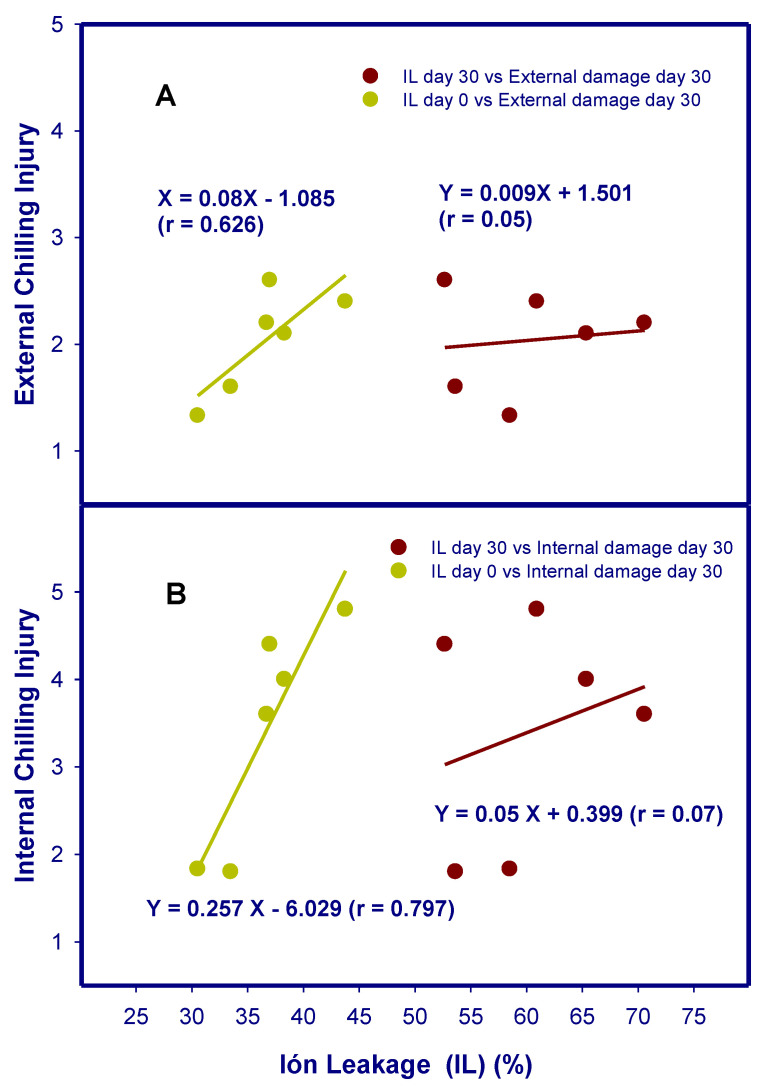
Correlation between external (**A**) and internal (**B**) chilling injury in pomegranate peels of different cultivars and ion leakage at harvest (green circle) and after storage for 30 d at 2 °C plus 2 d at 20 °C (red circle).

IL increased during storage in fruit peel for all cultivars. However, IL was not related to the CI sensitivity of cultivars. That is, the IL in pomegranate peel after storage was not correlated with internal (r = 0.07) or external (r = 0.05) CI indexes (Figure 3). On the contrary, the IL of the freshly harvested fruit (d 0) had a high positive correlation with the internal (r = 0.80) and external (r = 0.63) CI indexes in the peel after storage (Figure 3 and Table 2). Therefore, the IL of the peel of freshly harvested pomegranates could serve as an indicator of the state of the cell membranes and, in turn, predict the sensitivity of the fruit to suffer CI. On the other hand, the fatty acid composition of cell membranes of pomegranate peel was reported to change during storage at chilling temperatures, leading to a decrease in PUFA/SFA [9]. However, to date no pomegranate varietal comparative study has been carried out, in which CI symptoms have not been correlated with any increased IL or fatty acid composition.

**Figure 4:**
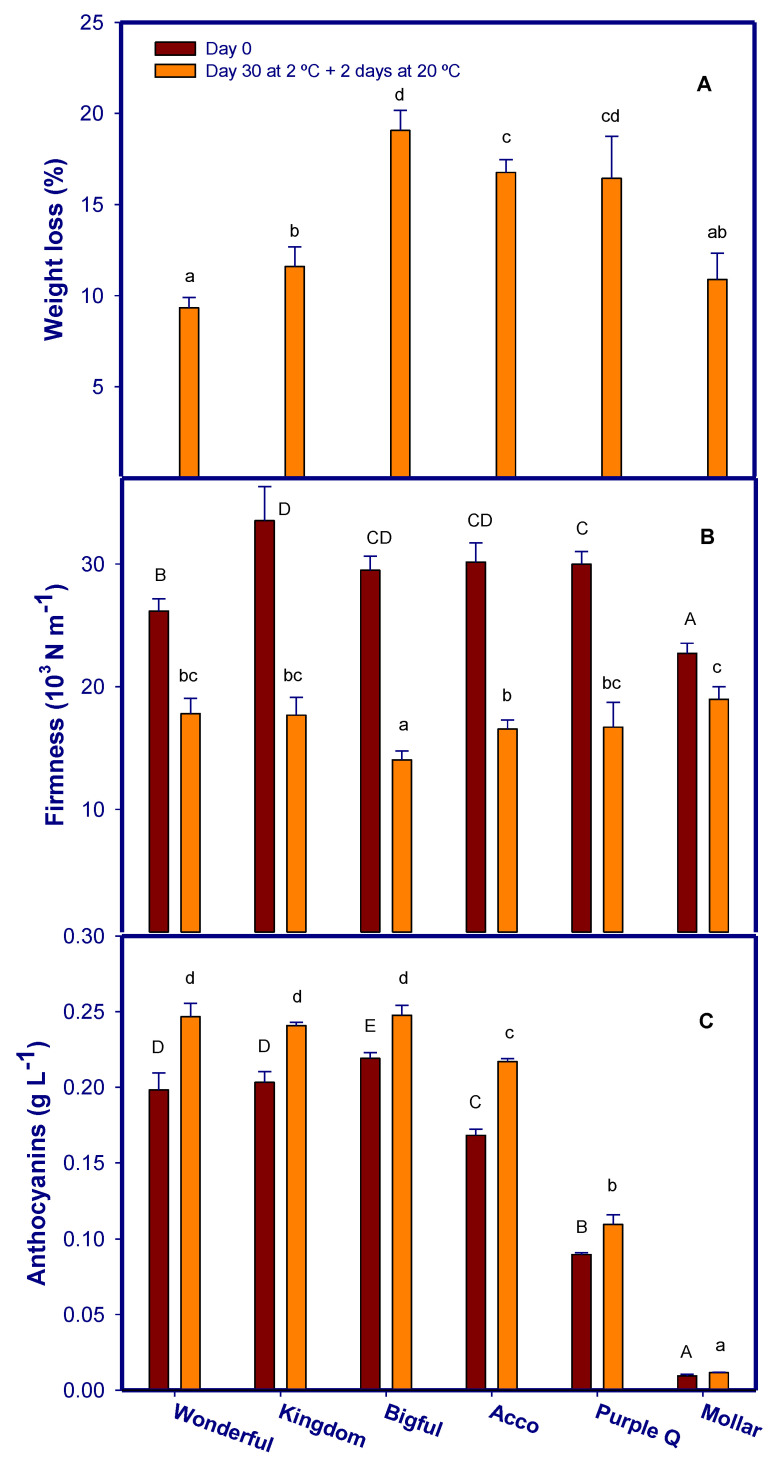
Weight loss (**A**), firmness of whole fruit (**B**) and total anthocyanins (**C**) of pomegranate cultivars at harvest (red bar) and after storage for 30 d at 2 °C plus 2 d at 20 °C (orange bar). Different tailored letters show significant differences (*p* < 0.05) between data at harvest. Different lowercase letters show significant differences (*p* < 0.05) between data after storage for 30 d at 2 °C plus 2 d at 20 °C. Data are the mean ± S.E. of determinations made in 3 replicates of 10 fruit.

The degree of weight loss in pomegranate fruits differs between cultivars, particularly when comparing their storage under different ambient conditions and temperatures. Factors that have a significant impact on moisture loss include fruit size, respiration rate, fruit peel–aril ratio, moisture content, and thickness of the peel [21]. These authors demonstrated that the moisture loss was significantly higher in medium-sized ‘Wonderful’ fruit compared to small-sized ‘Acco’ fruit. In our experiment, fruit weight loss ranged between 9.34 ± 0.56% for ‘Wonderful’ cultivar to 19.07 ± 1.09% for the ‘Bigful’ at the end of storage (Figure 4A). In this regard, the cultivars that showed the highest weight loss were not those that exhibited the most sensitivity to CI. Liu et al. [22] obtained similar results, since ‘Jingpitian’ and ‘Lishanhong’ pomegranate cultivars had the least weight loss and showed the highest CI symptoms when stored at 4 °C for 90 days, while ‘Tunisia soft seed’ cultivar showed high weight losses but very low incidence of CI. These authors attributed the weight losses of the pomegranates to the husk thickness. In addition, cultivars showing the highest weight loss also showed the lowest firmness, which was ~30% lower after 30 d of storage at 2 °C plus 2 d at 20 °C for ‘Acco’ and ‘Bigful’ cultivars (Figure 4B). Thus, a negative correlation (r = −0.63) was found between weight loss and firmness at the end of storage (Table 2). However, internal or external CI in pomegranate peel was not correlated to weight loss or firmness (Table 2). On the contrary when considering changes during storage in a particular cultivar, the increased weight and firmness losses during the storage were related to CI symptoms in pomegranate peel as reported for pomegranate ‘Mollar de Elche’ [5,8].

On the other hand, total aril anthocyanin content at harvest was different among the studied cultivars. The highest concentration was found in ‘Bigful’, ‘Wonderful’, and ‘Kingdom’ in concentrations of 0.22–0.20 g L^−1^, followed by ‘Acco’ (0.168 ± 0.004 g L^−1^) and ‘Purple Queen’ (0.089 ± 0.001 g L^−1^), while ‘Mollar de Elche’ had very low anthocyanin content (0.010 ± 0.001 g L^−1^) (Figure 4C). For all cultivars, the anthocyanin concentration increased up to ~25% during 30 d of storage at 2 °C plus 2 d at 20 °C (Figure 4C). Different papers have shown that anthocyanin content in pomegranate arils increased during cold storage [23] even if fruit are stored under chilling temperatures of 2 °C [12,24], as a consequence of an increased activity of phenylalanine ammonia lyase (PAL) and UDPglucose, flavonoid-3-O glucosyltransferase (GT), which are key enzymes in the phenolic and anthocyanin biosynthetic pathways [23].

Anthocyanin content in the arils at harvest had a high correlation with cultivar sensitivity to CI (external damage r = 0.75 and internal damage r = 0.89) (Table 3) as well as at the end of storage (external damage r = 0.85 and internal damage r = 0.94) (Table 2). Thus, the cultivars most sensitive to CI were those that had higher anthocyanin content at harvest and at the end of storage.

TSS content (Table 4) at harvest was in the range 13.28–16.10% in the different cultivars. ‘Mollar de Elche’, ‘Kingdom’, and ‘Wonderful’ cultivars showed the highest (>15), TSS concentration at harvest, 15.83 ± 0.72, 16.10 ± 0.13%, and 16.03 ± 0.16% respectively, ‘Acco’ and ‘Bigful’ had a TSS content of 14.23 ± 0.18% and 14.18 ± 0.15%, respectively, while ‘Purple Queen’ had 13.28 ± 0.09% of TSS. During storage, TSS did not change in ‘Mollar de Elche’, ‘Kingdom’, ‘Wonderful’, and ‘Bigful’ cultivars, while in ‘Acco’ and ‘Purple Queen’ it decreased. Sucrose, glucose, and fructose were the three sugars identified in all cultivars (Table 4). Sucrose concentration was lower than 0.7 g L^−1^ (accounting for 1% of total sugars), while glucose and fructose were found at much higher concentrations, ~50 g L^−1^ (each one accounting for ~49.0% of total sugar content). Glucose and fructose concentrations were within the range of values reported here in other pomegranate cultivars [2,5,25]. Sucrose concentration decreased in CI sensitive cultivars (‘Kingdom’ and ‘Wonderful’), while increases occurred in less sensitive cultivars (‘Bigful’, ‘Acco’, ‘Purple Queen’, and ‘Mollar de Elche’). Glucose and fructose decreased in all cultivars, except for ‘Kingdom’ and ‘Acco’ cultivars, in which no changes were observed (Table 4). In this sense, sucrose, at the end of storage, was negatively correlated with internal (r = −0.8) and external (r = −0.67) CI of pomegranate cultivars, while glucose concentration at the end of the experiment was positively correlated with internal (r = 0.71) and external (r = 0.75) CI indexes (Table 2). The accumulation of sugars (sucrose, glucose, or fructose) has been considered as a mechanism for protecting cell membranes and stabilizing macromolecular structures against CI [26,27]. However, in our case, only sucrose accumulated in the arils of the most tolerant pomegranate cultivars to CI, while glucose and fructose decreased or did not change with respect to the initial concentration. Thus, sucrose could be the most important sugar accounting for increasing CI tolerance in pomegranate cultivars.

TA of aril juice was different among cultivars (Table 4). The most acidic cultivars were ‘Kingdom’ and ‘Wonderful’ with TA higher than 12 g L^−1^, while ‘Acco’ and ‘Bigful’ cultivars had TA close to 7 g L^−1^. Finally, ‘Purple Queen’ and ‘Mollar de Elche’ cultivars had TA lower than 4 g L^−1^. Overall, among the cultivars chosen in this manuscript, those with the highest acidity, ‘Kingdom’ and ‘Wonderful’, were more sensitive to CI, than the low acidic cultivars, ‘Purple Queen’ and ‘Mollar de Elche’. According to Pearson correlations (Table 2 and Table 3), fruit TA at harvest was highly correlated (r = 0.91) with internal and external CI indexes, while fruit TA after 30 d at 2 °C plus 2 d at 20 °C of storage was adjusted to r = 0.87 for internal damage and r = 0.84 for external CI. In addition, no significant changes were observed in TA during storage for any of the cultivars (Table 4). In agreement with these results, Liu et al. [22] showed that pomegranate cultivars that were more susceptible to CI exhibited the highest TA over 90 days of storage at 4 °C. However, a more extensive study would be needed with a greater number of varieties and different cultivation conditions to assess if this relationship is general for all pomegranate cultivars.

Oxalic, citric, tartaric, malic, ascorbic, succinic, and fumaric organic acids were detected in all pomegranate cultivars (Table 4). Citric acid was the main organic acid in ‘Kingdom’, ‘Wonderful’, ‘Bigful’, and ‘Acco’ varieties in concentrations of 11.8, 9.7, 5.7, and 5.3 g L^−1^, respectively, which accounted for 84.7, 83.3, 62.3, and 60.7% of total organic acids, respectively. However, the citric acid concentration in ‘Purple Queen’ was of a similar fraction to ascorbic acid and were 34.8 and 30.2 % of total organic acid content, respectively. Finally, malic and citric acids were the main organic acids in ‘Mollar de Elche’ each one accounting for 30.7 and 27.9% (1.0 and 1.5 g L^−1^). The other organic acids were <20% of the total organic acids in all cultivars.

**Figure 5:**
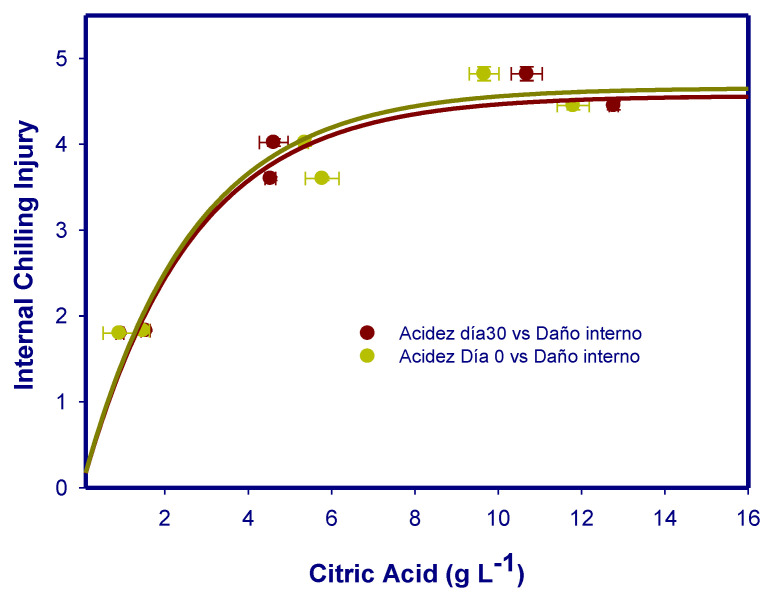
Nonlinear correlations between internal chilling injury on internal peel surface and citric acid concentration in juices at harvest (green circle) or and after storage (red circle). Data are the mean ± S.E. of determinations made in 3 replicates of 10 fruit.

Citric acid was the predominant organic acid in the more sensitive cultivars to CI. However, citric, malic, or ascorbic acids were in a low concentration and in a similar fraction in the less sensitive cultivars to CI. In addition, in the most CI sensitive cultivars malic acid was degraded during cold storage. However, in the less CI sensitive cultivars to CI (‘Acco’ and ‘Mollar de Elche’), malic acid did not change during storage. On the other hand, the concentration of oxalic acid was higher and increased during storage in the cultivars more resistant to CI. According to the Pearson correlations (Table 2 and Table 3), the malic and oxalic acid concentrations, both at harvest and at the end of storage, exhibited high negative correlations (r > −0.8) with external and internal CI damage. However, citric acid concentrations, both at harvest and at the end of storage, showed high positive correlations (r ~ 0.9) with external and internal CI. In this sense, CI of the peel internal surface had high positive correlations by means of exponential type curves of the maximums with the citric acid concentrations, both at the time of harvest (y = 4.65 × (1 − exp(−3.85 × x) r = 0.98) and at the end of storage (y = 4.56 × (1 − exp(−3.82 × x) r = 0.96) (Figure 5).

Juice leakage from the arils could lead to the internal peel CI being manifested by browning spots. In fact, Fawole et al. [28] and Ramezanian et al. [29] reported that the leakage or loss of juice from the arils is due to integrity loss of the aril cell membranes which leads to increases in membrane permeability. Organic acids develop an important role in regulating osmotic pressure, pH homeostasis, and stress resistance in fruit cells [30]. The permeability loss and fluidity change of cell membranes, including plasmatic and organelles membranes, such as tonoplast, are a consequence of CI. The increase in permeability of all cell membranes leads to the leakage of solutes such as protons or protonated forms of organic acids, towards the cell wall [31]. The high concentrations of sugars, organic acids, and inorganic ions inside the vacuole generate high osmotic pressure that leads to a strong negative water potential [32]. Accordingly, ‘Wonderful’ and ‘Kingdom’, with higher content of organic acids and other solutes such as anthocyanins would have higher osmotic pressure and lower water potential than less CI sensitive cultivars. Consequently, the aril cell membranes of CI susceptible cultivars, with a higher internal pressure in the vacuoles, would have a greater leakage of solutes through both the tonoplast and the plasmalemma leading quickly to contact with placental tissue and the internal peel surface of pomegranates, since arils are composed of a single layer of parenchymatic cells between the seed and its surface [33]. On the other hand, the oxalic acid role on defense mechanisms against biotic and abiotic stress of the fruit has been studied, and one of these effects is increase of the antioxidant systems as has been reported in lemon fruit [34]. In addition, treatments with oxalic acid have improved fruit quality at harvest [20] and during postharvest and have reduced chilling injury pomegranate [35]. In this sense, the accumulation of oxalic acid in ‘Mollar de Elche’ and ‘Purple Queen’ cultivars after 30 d of storage at 2 °C plus 2 d at 20 °C could reduce their CI sensitivity.

Regarding mineral composition in the peel of pomegranates, K (14.1–7.7 g kg^−1^) and Ca (3.6–2.7 g kg^−1^) were the major elements, both accounting for approximately ~98% of the total quantified minerals (Table 5). Na (0.72–0.17 g kg^−1^) and Mg (0.53–0.32 g kg^−1^) accounted for ~1% of the total minerals, while Cu, Fe, Mn, and Zn were found at much lower concentration (<0.014 g kg^−1^).

The sum of total mineral was positively correlated with the sensitivity of the pomegranates to CI (r = 0.76 for the external damage and r = 0.69 for the internal damage). Thus, the ‘Wonderful’ and ‘Kingdom’ cultivars had a total mineral content close to 17 g kg^−1^ while the remaining cultivars had a content lower than 15 g kg^−1^. Potassium was the only mineral that was positively correlated with external (r = 0.71) and internal (r = 0.66) CI damage. The remaining quantified minerals were not correlated with CI symptoms.

There is extensive information on calcium levels in fruit and the occurrence of CI in several fruit species [36] and even in pomegranate [29]. In the present experiment, all the analyzed cultivars were grown in the same field and under the same cultivation system while the concentration of calcium in the skin was similar in all cultivars and did not correlate with the appearance of CI (r = 0.1) (Table 3). However, according to Pearson correlation, the Ca/K ratio was negatively correlated with the sensitivity of the pomegranate cultivars to CI (r = −0.69) for external damage and r = −0.66 for internal damage). The balance between calcium and potassium (Ca/K) is considered to be a good indicator of sensitivity to biotic and abiotic disorder in plant tissues [37]. Several works have shown that a low ratio of Ca/K concentration is related to a high incidence of various disorders such as bitter pit in apple [38], appearance of water-soaked in peaches [39], and water-core in pears and apples [40]. In addition, the Ca/K ratio has been postulated as a good indicator to ensure post-harvest fruit quality [41]. High concentrations of K can have antagonist effects to Ca in vegetable tissues leading to increased tissue sensitivity to disease and disorder incidence [42]. K and Mg compete with Ca for binding sites at the cell wall and plasma membrane, which can negatively affect membrane stability and increase susceptibility to Ca deficiency disorders [43,44].

**Figure 6:**
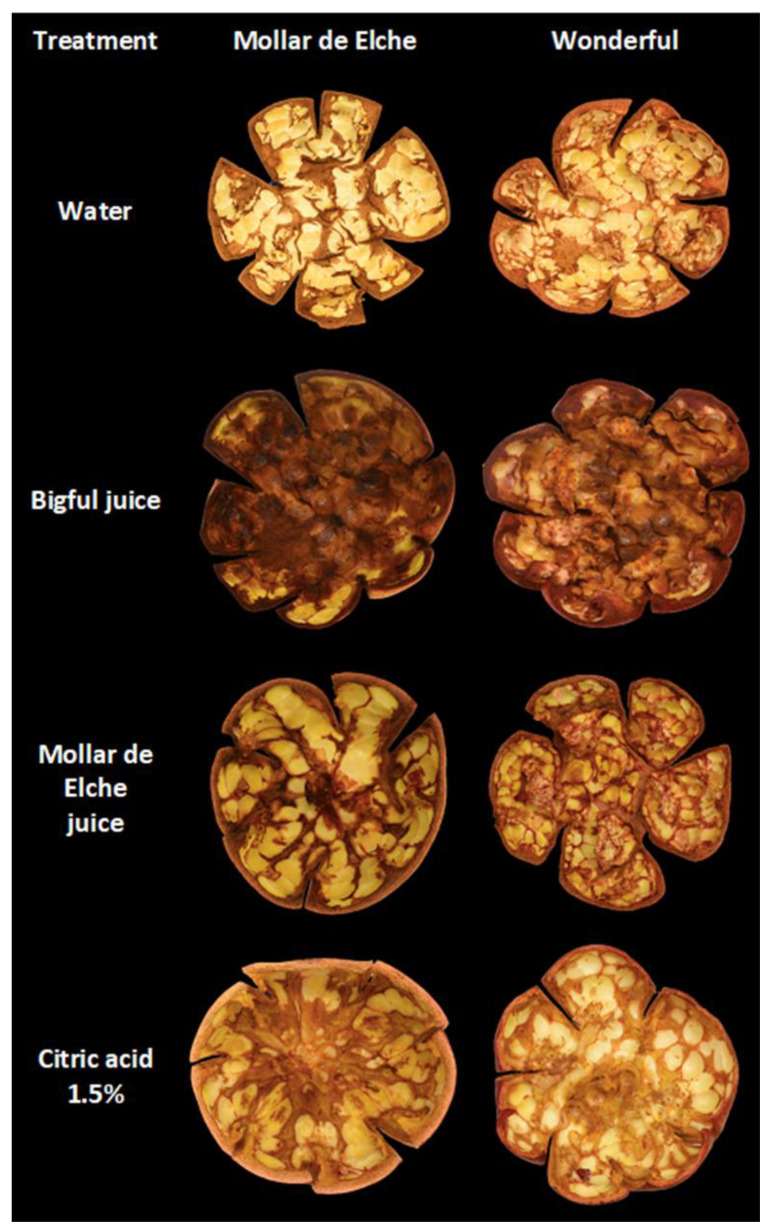
Internal aspect of ‘Wonderful’ and ‘Mollar de Elche’ peels treated with water, ‘Bigful’ or ‘Mollar de Elche’ juices, and citric acid at 1.5%, after 15 d at 2 °C.

According to the CI sensitivity test (Figure 6), citric acid and water treatments of ‘Mollar de Elche’ and ‘Wonderful’ peels led to a similar internal damage appearance of both cultivars. In both cases, only the browning related to the physical breakage of the tissues during manual peeling occurred. However, the inner surface of the pomegranate peel treated with ‘Bigful’ pomegranate juice caused a strong browning, while the pomegranates treated with ‘Mollar de Elche’ pomegranate juice were only slightly browned.

Molla et al. [45] reported that in pomegranate suffering from CI, aril red color decreased, peel tissue became spongy, and cell membrane permeability increased. In this sense, the juices from the damaged arils could leak onto the white tissues of the peel and stimulate the browning reactions. Accordingly, Liu et al. [22] showed that ‘Jingpitian’ and ‘Lishanhong’ cultivars, which are susceptible to CI, displayed notable browning and changes in husk microstructure during storage at 4 °C for 90 d, showing widening of microcracks, obstruction of lenticels, and disintegration of the external wax layer.

As shown in Figure 6, citric acid did not induce browning of the internal surface of pomegranate peel. The high polyphenol (anthocyanin) concentration of ‘Bigful’ juice would interact with the internal peel surface and carpel tissues leading to browning, while ‘Mollar de Elche’ juice which has low anthocyanin concentration, did not cause such high browning. In addition, polyphenols and flavonoids, apart from anthocyanins, would also migrate towards the inner peel surface reaching the cell skin and leading to browning by PPO enzyme which has a high activity in peel tissues [46]. On the other hand, it should be added that the peel of the cultivars more sensitive to cold damage showed a greater electrolyte leakage at the time of harvest, which would facilitate the enzymatic browning reactions of phenolics coming from the arils by peel polyphenol oxidase (PPO), peroxidase (POD), or anthocyanase [47].

## 4. Conclusions

From the obtained results we can conclude that there is great variability in the sensitivity to the appearance of CI among the different cultivars of pomegranates. ‘Kingdom’ cultivar showed the highest CI index while the least sensitive was ‘Mollar de Elche’. This variability is not only due to changes in cell membrane permeability, but also to the solute content in the vacuoles (anthocyanins, sugars, and organic acids, in particular citric acid) which can migrate to the inner surface of the peel causing browning reactions. In other words, cultivars with a high content of organic acids, sugars, and anthocyanins in their arils, a low ratio of Ca/K in their peel, and high membrane permeability at harvest would be the most susceptible to CI damage during storage.

## Figures and Tables

**Table 1:** Characteristic of pomegranate cultivars at harvest.

		Wonderful	Kingdom	Bigful	Acco	Purple Queen	Mollar
Diameter (mm)		105.80 ± 10.67	99.40 ± 9.10	90.11 ± 1.11	81.16 ± 1.78	79.19 ± 1.08	112.79 ± 3.02
Weight (g)		423.50 ± 14.53	433.71 ± 14.35	333.26 ± 6.49	311.57 ± 6.20	255.05 ± 3.99	435.07 ± 13.11
Color	L	51.94 ± 2.33	52.36 ± 1.75	54.38 ± 0.94	52.88 ± 0.77	52.49 ± 0.78	70.17 ± 1.93
	a	40.55 ± 2.42	41.61 ± 2.22	47.73 ± 0.95	48.68 ± 0.90	45.97 ± 0.91	16.11 ± 1.74
	b	26.33 ± 0.68	27.93 ± 0.51	26.61 ± 0.49	27.18 ± 0.40	26.60 ± 0.51	37.92 ± 0.61

Data are the mean ± SE of 3 replicates of 10 fruit.

**Table 2:** Pearson correlation between dependent variables of fruit after storage for 30 d at 2 °C plus 20 d at 20 °C.

	TSS	TA	Ext D	Int D	IL	WL	Firm.	ACNs	Oxalic	Citric	Tartaric	Malic	Ascorbic	Succinic	Fumaric	Sucr.	Gluc.	Fruct.	Ca	Cu	Fe	K	Mg	Mn	Na	Zn	ΣMin.
TA	0.76 ***	--																									
Ext D	0.68 ***	0.84 ***	--																								
Int D	0.56 **	0.87 ***	0.93 ***	--																							
IL	−0.54 ***	−0.09	0.05	0.07	--																						
WL	−0.7 ***	−0.43 **	−0.28	−0.18	0.36 *	--																					
Firm.	0.47 **	0.33 *	0.5 ***	0.49 *	0.09	−0.63 ***	--																				
ACNs	0.39 *	0.80 ***	0.85 ***	0.94 ***	0.27	−0.1	0.26	--																			
Oxalic	−0.36	−0.74 ***	−0.88 ***	−0.94 ***	−0.26	0.01	−0.41 *	−0.94 ***	--																		
Cítric	0.82 ***	0.95 ***	0.87 ***	0.87 ***	−0.25	−0.4	0.47 *	0.84 ***	−0.73 ***	--																	
Tartaric	0.71 ***	0.75 ***	0.59 **	0.62 ***	−0.35	−0.55 **	0.50 *	0.57 **	−0.45 *	0.75 ***	--																
Málic	−0.62 ***	−0.91 ***	−0.84 ***	−0.91 ***	0.06	0.19	−0.29	−0.94 ***	0.81 ***	−0.94 ***	−0.68 ***	--															
Ascorbic	−0.67 ***	−0.24	−0.02	0.10	0.60 **	0.74 ***	−0.38	0.21	−0.33	−0.24	−0.45 *	0.01	--														
Succinic	−0.85 ***	−0.58 **	−0.34	−0.28	0.56 **	0.75 ***	−0.52 **	−0.18	0.03	−0.59 **	−0.7 ***	0.38	0.87 ***	--													
Fumaric	−0.31	−0.01	0.19	0.24	0.51 *	0.36	−0.02	0.27	−0.39	−0.02	−0.16	−0.19	0.64 ***	0.53 **	--												
Sucr.	−0.48 *	−0.83 ***	−0.67 ***	−0.8 ***	0.04	0.07	−0.05	−0.9 ***	0.72 ***	−0.86 ***	−0.6 **	0.94 ***	−0.05	0.32	−0.12	--											
Gluc.	0.8 ***	0.78 ***	0.75 ***	0.71 ***	−0.29	−0.42 *	0.59 **	0.56 **	−0.51 *	0.83 ***	0.64 ***	−0.72 ***	−0.33	−0.54 **	−0.06	−0.51 **	--										
Fruct.	0.74 ***	0.51 *	0.48 *	0.38	−0.42 *	−0.53 **	0.62 ***	0.17	−0.15	0.56 **	0.49 **	−0.37	−0.52 *	−0.56 **	−0.19	−0.13	0.90 ***	--									
Ca	0.01	−0.06	0.05	0.12	0.03	−0.27	0.41	−0.07	−0.01	−0.11	0.10	0.03	−0.03	−0.06	0.18	0.22	0.11	0.21	--								
Cu	0.36	0.57 **	0.70 ***	0.58 **	0.01	−0.02	0.17	0.63 **	−0.65 **	0.58 *	0.19	−0.53 *	0.16	−0.02	−0.09	−0.48	0.43	0.19	−0.32	--							
Fe	−0.17	0.10	0.42	0.35	0.53 *	0.39	0.09	0.37	−0.54 *	0.06	−0.09	−0.17	0.51 *	0.52	0.28	−0.05	0.03	−0.15	−0.15	0.55 **	--						
K	0.87 ***	0.81 ***	0.71 ***	0.66 **	−0.51 *	−0.42	0.60 **	0.51 *	−0.48 *	0.80 ***	0.69 **	−0.63 **	−0.39	−0.65 **	−0.15	−0.51 *	0.84 ***	0.74 ***	0.08	0.42	−0.07	--					
Mg	0.01	−0.09	−0.04	0.02	0.049	−0.47 *	0.45	−0.13	0.08	−0.12	0.16	0.09	−0.19	−0.16	−0.01	0.24	0.02	0.14	0.87 ***	−0.37	−0.2	−0.06	--				
Mn	−0.42	−0.06	0.26	0.32	0.70 ***	0.38	0.18	0.29	−0.48	−0.13	−0.13	−0.07	0.70 ***	0.56 *	0.61 **	0.01	−0.15	−0.3	0.44	0.1	0.64 **	−0.21	0.33	--			
Na	−0.05	0.08	0.33	0.18	0.21	0.30	−0.11	0.24	−0.25	0.10	−0.13	−0.15	0.30	0.25	−0.04	−0.07	0.10	−0.03	−0.21	0.49 **	0.54 **	−0.09	−0.29	0.20	--		
Zn	0.10	0.33	0.44	0.40	0.01	0.46	−0.17	0.45 *	−0.47 *	0.31	0.08	−0.34	0.28	0.16	−0.02	−0.36	0.24	0.03	−0.45 *	0.67 **	0.54	0.31	−0.68 **	0.10	0.48 *	--	
ΣMin.	0.83 ***	0.78 ***	0.76 ***	0.69 ***	−0.44	−0.42	0.66 **	0.51 *	−0.51 *	0.76 ***	0.67 **	−0.62 **	−0.33	−0.6 **	−0.11	−0.4	0.85 ***	0.76 ***	0.29	0.42	−0.01	0.96 ***	0.11	−0.06	0.04	0.26	--
Ca/K	−0.84 ***	−0.77 ***	−0.69 ***	−0.66 **	0.48 *	0.46 *	−0.66 **	−0.61 **	0.46 *	−0.74 ***	−0.69 ***	0.59 **	0.38	0.64 ***	0.1	0.39	−0.83 ***	−0.76 ***	−0.29	−0.34	0.1	−0.97 ***	−0.13	0.11	0.13	−0.19	−0.98 ***

Red letters show the correlation between external and internal CI indexes and dependent variables. TSS: Total Soluble Solids, TA: Titratable Acidity, Ext D or Int D: external or internal peel CI index, IL: Ion Leakage, WL: Weight Loss, ACNs: Anthocyanins, Firm.: Firmness, Sucr.: Sucrose, Gluc.: Glucose, Fruct.: Frutose, ΣMin.: Mineral Sum. *** Significant difference at *p* < 0.001. ** Significant difference at *p* < 0.01. * Significant difference at *p* < 0.05.

**Table 3:** Pearson correlation between dependent variables of fruit at harvest and external and internal damage of fruit storage for 30 d at 2 °C plus 20 d at 20 °C.

	Pomegranate Variables at Harvest														
30 d at 2 °C	TSS	TA	ILd0	Firm.	ACNs	Oxalic	Citric	Tartaric	Malic	Ascorbic	Succinic	Fumaric	Sucr.	Gluc.	Fruct.
Ext D	0.64 ***	0.91 ***	0.63 ***	−0.004	0.75 ***	−0.91 ***	0.90 ***	0.34	−0.84 ***	−0.02	−0.20	0.04	−0.59 **	0.11	−0.06
Int D	0.59 **	0.91 ***	0.80 ***	−0.001	0.89 ***	−0.92 ***	0.89 ***	0.31	−0.90 ***	0.06	−0.19	0.13	−0.66 ***	0.05	−0.12
ILd30	−0.39 *	−0.02	0.16	0.17	0.42 *	−0.25	−0.19	−0.50 *	0.13	0.60 **	0.58 *	0.58 *	0.28	−0.56 *	−0.58 *

Ext D or Int D: external or internal damage on peel, ILd0: Ion Leakage d 0, ILd30: Ion Leakage d 30, TSS: Total Soluble Solids, TA: Titratable Acidity, ACNs: Anthocyanins, Firm: Firmness, Sucr: Sucrose, Gluc: Glucose, Fruct: Fructose. *** Significant difference at *p* < 0.001. ** Significant difference at *p* < 0.01. * Significant difference at *p* < 0.05.

**Table 4:** Total soluble solids, titratable acidity (TA), organic acid and sugar contents in aril juice of different pomegranate cultivars.

Time and Temp.	Cultivar	TSS (%)	TA (g L^−1^)	Oxalic (10^−3^ g L^−1^)	Citric (g L^−1^)	Tartaric (10^−3^ g L^−1^)	Malic (g L^−1^)	Ascorbic (g L^−1^)	Succinic (g L^−1^)	Fumaric (10^−3^ g L^−1^)	Sucrose (g L^−1^)	Glucose (g L^−1^)	Fructose (g L^−1^)
At harvest	Wonderful	16.10 ± 0.13 cA	12.1 ± 0.3 cA	3.8 ± 0.5 aA	9.7 ± 0.2 dA	21.0 ± 1.8 bA	0.63 ± 0.04 aA	0.77 ± 0.01 aA	0.5 ± 0.09 aA	0 aA	0.31 ± 0.01 aA	50.9 ± 01.5 abA	49.4 ± 1.4 abA
	Kingdom	16.03 ± 0.16 cA	13.8 ± 1.2 dA	5.6 ± 0.1 aA	11.8 ± 0.3 eA	40.3 ± 3.1 bA	0.60 ± 0.01 aA	0.92 ± 0.01 aA	0.45 ± 0.09 aA	0 aA	0.29 ± 0.01 aA	49.2 ± 0.7 abA	47.7 ± 0.6 abA
	Bigful	14.23 ± 0.18 bA	7.3 ± 0.1 bA	4.6 ± 0.1 aA	5.4 ± 0.1 bA	0 aA	0.73 ± 0.02 bA	1.69 ± 0.03 cA	0.81 ± 0.03 bA	79.6 ± 4.8 bA	0.44 ± 0.06 bA	48.5 ± 1.0 abA	47.3 ± 1.0 abA
	Acco	14.18 ± 0.15 aA	7.1 ± 0.4 bA	3.4 ± 0.2 aA	6.0 ± 0.2 cA	0 aA	0.57 ± 0.03 aA	1.97 ± 0.12 cA	1.13 ± 0.02 bA	132.2 ± 5.6 dA	0.40 ± 0.13 bA	44.4 ± 0.3 aA	42.9 ± 0.4 aA
	Purple Q	13.28 ± 0.09 aA	3.9 ± 0.2 aA	5.7 ± 0.3 cA	1.5 ± 0.4 aA	0 aA	0.86 ± 0.02 cA	1.31 ± 0.01 bA	0.49 ± 0.02 aA	101.2 ± 0.6 cA	0.53 ± 0.01 cA	44.1 ± 0.9 abA	44.7 ± 1.1 abA
	Mollar	15.83 ± 0.72 bA	3.6 ± 0.2 aA	4.1 ± 0.1 bA	0.9 ± 0.1 aA	45.6 ± 01.2 cA	1.00 ± 0.15 cA	0.67 ± 0.02 aA	0.59 ± 0.02 aA	0 aA	0.66 ± 0.03 dA	51.4 ± 1.3 bA	53.4 ± 1.1 bA
30 d at 2 °C plus 2 d at 20 °C	Wonderful	15.78 ± 0.04 dA	13.4 ± 1.1 cA	3.5 ± 0.2 aA	10.7 ± 0.3 cA	6.5 ± 2.1 bB	0 aB	0.79 ± 0.02 aA	0.05 ± 0.01 aB	48.4 ± 1.8 bB	0 aB	48.2 ± 0.3 bcB	46.7 ± 0.4 abB
	Kingdom	16.30 ± 0.12 eA	15.7 ± 1.1 dA	5.7 ± 0.1 aA	12.7 ± 0.1 dA	5.0 ± 3.8 abB	0 aB	0.95 ± 0.05 abA	0.09 ± 0.01 aB	57.5 ± 2.6 cB	0 aB	49.5 ± 1.3 cA	48.1 ± 1.1 bA
	Bigful	14.13 ± 0.10 bA	7.5 ± 0.1 bA	5.7 ± 0.1 aA	4.6 ± 0.2 bA	8.1 ± 5.7 abB	0.47 ± 0.04 bB	1.66 ± 0.09 cA	1.17 ± 0.2 dB	84.1 ± 5.7 dB	0.59 ± 0.04 bB	46.2 ± 0.7 abcB	45.6 ± 0.7 abB
	Acco	13.82 ± 0.06 abB	6.9 ± 0.1 bA	5.1 ± 0.5 aA	4.5 ± 0.1 bA	6.2 ± 4.3 abB	0.49 ± 0.02 bB	1.69 ± 0.08 cB	1.27 ± 0.1 dB	88.3 ± 1.7 dB	0.58 ± 0.05 bB	43.8 ± 0.4 abA	43.3 ± 0.3 aA
	Purple Q	12.08 ± 0.07 aB	3.9 ± 0.6 aA	22.2 ± 6.5 bA	1.9 ± 0.6 aA	1.7 ± 1.1 aB	0.56 ± 0.12 cB	1.12 ± 0.22 bA	0.85 ± 0.02 cB	52.8 ± 1.5 cB	0.59 ± 0.09 gB	35.3 ± 3.5 aB	35.7 ± 7.1 aB
	Mollar	14.92 ± 0.23 cA	3.6 ± 0.1 aA	34.6 ± 0.1 bA	0.9 ± 0.1 aA	8.4 ± 3.0 abB	1.08 ± 0.02 dA	0.65 ± 0.01 aB	0.44 ± 0.01 bB	40.0 ± 5.4 aB	1.43 ± 0.02 cB	44.6 ± 0.7 abB	47.1 ± 0.7 abB

Data are the mean ± SE of 3 replicates of 10 fruit. Different tailored letters show significant differences (*p* < 0.05) between data at harvest and after storage for each cultivar and dependent variable. Different lowercase letters show significant differences (*p* < 0.05) among cultivars for each dependent variable and storage time.

**Table 5:** Mineral contents in pomegranate peel after storage at 2 °C plus 20 d at 20 °C.

Cultivar	Ca (g kg^−1^)	Cu (10^−3^ g kg^−1^)	Fe (10^−3^ g kg^−1^)	K (g kg^−1^)	Mg (g kg^−1^)	Mn (10^−3^ g kg^−1^)	Na (g kg^−1^)	Zn (10^−3^ g kg^−1^)	Σ Minerals (g kg^−1^)	Ca/K
Wonderful	3.2 ± 0.1 b	3.1 ± 0.4 b	4.7 ± 0.5 c	12.8 ± 0.7 b	0.53 ± 0.02 a	5.4 ± 0.3 c	0.26 ± 0.09 ab	9.4 ± 1.4 bc	16.8 ± 0.8 a	0.24 ± 0.01 d
Kingdom	2.6 ± 0.1 c	4.0 ± 0.4 a	6.0 ± 1.2 bc	14.1 ± 0.3 c	0.32 ± 0.02 d	3.7 ± 0.1 e	0.57 ± 0.06 c	13.8 ± 2.4 a	17.6 ± 0.6 a	0.18 ± 0.03 e
Bigful	3.4 ± 0.3 ba	2.7 ± 0.2 b	7.8 ± 1.8 b	10.6 ± 0.2 b	0.40 ± 0.03 c	6.9 ± 0.4 b	0.47 ± 0.06 bc	13.5 ± 2.5 a	14.9 ± 0.6 b	0.32 ± 0.01 c
Acco	3.1 ± 0.2 b	3.8 ± 0.6 a	10.2 ± 2 a	9.1 ± 0.8 d	0.44 ± 0.01 c	7.7 ± 0.5 a	0.72 ± 0.14 c	11.6 ± 1.2 ab	13.3 ± 0.5 bcd	0.34 ± 0.01 bc
Purple Q	2.7 ± 0.4 cb	2.3 ± 0.4 cb	3.7 ± 0.5 d	7.7 ± 0.7 e	0.35 ± 0.05 dc	3.9 ± 0.4 de	0.38 ± 0.09 b	10.6 ± 1.7 ab	11.2 ± 0.9 d	0.35 ± 0.1 ab
Mollar	3.6 ± 0.1 a	2.1 ± 0.1 c	3.9 ± 0.4 cd	9.8 ± 0.5 d	0.50 ± 0.02 b	4.4 ± 0.3 b	0.17 ± 0.03 a	7.3 ± 0.6 c	14.0 ± 0.6 c	0.37 ± 0.01 a

Data are the mean ± SE of 3 replicates of 10 fruit. Different letters show significant differences (*p* < 0.05) among cultivars for each dependent variable.

## Data Availability

Data is contained within the article.
